# Identification of critical amino acid residues in the regulatory N-terminal domain of PMEL

**DOI:** 10.1038/s41598-021-87259-y

**Published:** 2021-04-08

**Authors:** Susan M. Mitchell, Morven Graham, Xinran Liu, Ralf M. Leonhardt

**Affiliations:** 1 Department of Immunobiology, Yale University School of Medicine, 300 Cedar Street, New Haven, CT 06519 USA; 2 Department of Cell Biology, Yale University School of Medicine, 300 Cedar Street, New Haven, CT 06519 USA; 3 Boehringer Ingelheim RCV GmbH & Co KG, Cancer Immunology and Immune Modulation, Dr. Boehringer Gasse 5-11, 1121 Vienna, Austria

**Keywords:** Lysosomes, Multivesicular bodies, Protein aggregation

## Abstract

The pigment cell-specific protein PMEL forms a functional amyloid matrix in melanosomes onto which the pigment melanin is deposited. The amyloid core consists of a short proteolytic fragment, which we have termed the core-amyloid fragment (CAF) and perhaps additional parts of the protein, such as the PKD domain. A highly O-glycosylated repeat (RPT) domain also derived from PMEL proteolysis associates with the amyloid and is necessary to establish the sheet-like morphology of the assemblies. Excluded from the aggregate is the regulatory N-terminus, which nevertheless must be linked *in cis* to the CAF in order to drive amyloid formation. The domain is then likely cleaved away immediately before, during, or immediately after the incorporation of a new CAF subunit into the nascent amyloid. We had previously identified a 21 amino acid long region, which mediates the regulatory activity of the N-terminus towards the CAF. However, many mutations in the respective segment caused misfolding and/or blocked PMEL export from the endoplasmic reticulum, leaving their phenotype hard to interpret. Here, we employ a saturating mutagenesis approach targeting the motif at single amino acid resolution. Our results confirm the critical nature of the PMEL N-terminal region and identify several residues essential for PMEL amyloidogenesis.

## Introduction

PMEL is a pigment cell protein expressed in melanocytes and retinal pigment epithelium^1^. In these cells, it forms a functional amyloid matrix in melanosomes^2^, which are lysosome-related organelles^3^. The amyloid matrix serves for the deposition of the pigment melanin^2^. Sequestering highly oxidative reaction intermediates derived from the melanin synthesis pathway^4^ on PMEL fibrillar aggregates likely prevents their toxic leakage out of melanosomes and may accelerate melanin formation^2,5^. Mutations in PMEL are associated with pigmentation disorders and/or impairments of eye development in various species including fish^6^, chickens^7^, mice^8,9^, horses^10^, cattle^11^, and dogs^12^. In humans, mutations in PMEL were recently linked to pigment dispersion syndrome and pigmentary glaucoma^13^. Gain-of-function mutations in PMEL, perhaps driving toxic misaggregation, have a tendency to lead to stronger pigmentation phenotypes than loss-of-function mutations^14^. Besides its well-established role in pigmentation biology, PMEL is also an abundant tumor antigen highly expressed in human melanoma^15^. Epitopes derived from the protein are promising targets for cancer immunotherapy^16,17^. Recent reports implicate PMEL aggregation in melanoma progression^18^.

As a type I transmembrane protein, newly synthesized PMEL is inserted into the endoplasmic reticulum (ER) membrane as a ~ 100 kDa species called P1. P1 is rapidly exported from the ER and undergoes extensive O-glycosylation, particularly in the repeat (RPT) domain, as well as maturation of various N-glycans in the Golgi^19,20^. This gives rise to a ~ 120 kDa form of PMEL named P2, which is subsequently cleaved by proprotein convertases^21^ during passage through secretory compartments^22,23^. This cleavage results in a ~ 90 kDa N-terminal Mα fragment and a ~ 28 kDa C-terminal, membrane-tethered Mβ fragment. These fragments remain linked to each other by a disulfide bond involving cysteine-301 in a PMEL cross-dimer^24^. After arrival at the cell surface, the protein is endocytosed via a di-leucine-based endocytic motif and delivered into stage I melanosomes^25^, which represent a specialized early endosomal compartment^26^. Via a lumenal determinant, PMEL is then sorted into domains that invaginate inwards from the limiting melanosomal membrane in a CD63-dependent manner. These invaginations eventually pinch off, and form intralumenal vesicles (ILVs)^26–28^. Subsequently, the protease BACE2 cleaves within Mβ, which releases the Mα fragment, probably still disulfide-linked to a small piece of Mβ^29^, from the ILV surface and into the lumen of the developing melanosome^18,30^. We provide a detailed overview of the PMEL processing pathway in Suppl. Fig. S1.

Mα consists of four domains: the N-terminal fragment (NTF) (amino acid (aa) 26 to ~ 147), the core amyloid fragment (CAF) (aa ~ 148 to ~ 223), the polycystic kidney disease-like (PKD) domain (aa 255 to 297), and the RPT domain (302–457)^31^ (see also Suppl. Fig. S1). There is a lot of controversy in the field as to which of these domains carries the amyloid-forming activity in vivo. The PKD domain is essential for PMEL function^27^ and long known to be a constituent of the amyloid^19,29,32^. However, lack of proper reagents and technical difficulties have hampered its study. Hence, the biochemistry of the PKD domain is still understudied, even though it contains remarkable amyloidogenic activity in vitro^33^ and, thus, may contribute directly to fibril formation.

It is also long known that the highly O-glycosylated RPT domain is associated with melanosomal amyloid^34^. Because bacterially produced, unglycosylated RPT domain was shown to aggregate in vitro, it was originally discussed as the leading candidate for forming the amyloid core^35^. However, there exists no evidence that the RPT domain forms amyloid in vivo, and the dense O-glycosylation of this fragment in vertebrate cells^20,36^, would likely shield necessary protein–protein interactions. Moreover, amyloid formed in vitro by artificial unglycosylated RPT domain fragments dissolves at neutral pH^37^. This is non-physiological, because pH neutralization during melanosome development^38–40^ is required for tyrosinase to be enzymatically active and produce melanin^41,42^. As a consequence, acidic pH-addicted RPT fibrils would be predicted to fall apart in vivo in the melanosome right in the moment when they are most needed, at the time when the pigment is beginning to be synthesized.

The RPT domain is the only major PMEL domain whose sequence is not conserved in evolution, although RPT domains from all vertebrates are predicted to be highly O-glycosylated^36,43^. Moreover, the RPT domain is not required for PMEL amyloid formation in vitro^33^. Recently, we demonstrated that the RPT domain is also dispensable for amyloid formation in vivo in living human cells^44^. Our laboratory extensively characterized PMEL amyloid formed in the absence of the RPT domain in situ in melanosomes by electron tomography^36^. This work demonstrated that if the RPT domain is lacking, the characteristic sheet morphology^45^ of the aggregated matrix collapses^36^. This phenomenon is dependent on RPT domain-associated O-glycans, the only conserved feature of this domain, but it is not dependent on the RPT domain amino acid sequence^36^. Taken together, the RPT domain is almost certainly not the amyloid-forming domain of the PMEL protein. Instead, it represents an accessory module that likely associates peripherally with the fibrillar matrix and shapes its morphology.

In search of the PMEL domain driving amyloidogenesis in melanosomes another candidate has emerged: the ~ 8 kDa CAF. The first evidence pointing in this direction was the demonstration by mass spectrometry that amyloid cores of in vitro-aggregated PMEL fragments contain sequences derived from the CAF^33^. Moreover, fibrillar fractions from melanoma cells contain high levels of this fragment^31,33,46^. Alanine-scanning mutagenesis revealed numerous residues that when mutated fully suppressed or substantially reduced amyloid formation in living human cells, while the precursors of these mutants often behaved normally with respect to trafficking and processing up until arriving in melanosomes^31^. Various amyloid prediction algorithms identified amyloidogenic regions within the fragment^31^. The CAF is highly conserved in evolution, and many of the residues identified as most critical for amyloidogenesis display 100% conservation in species as diverse as mammals, birds, reptiles, amphibians, and fish^31^. Across these species, structure modeling predicts a β-solenoid fold for the fragment, and β-solenoids can be building blocks of amyloids^47^. Strikingly, the prediction of the β-solenoid structure breaks down when mutations which disrupt amyloid formation in vivo are introduced into the sequence^31^. Thus, we conclude that the CAF, not the RPT domain, is the major driver of amyloid formation in melanosomes.

First mechanistic insights have emerged into how PMEL aggregation may be regulated in vivo. Once it is proteolytically released from the ILV membrane, the Mα fragment undergoes a further cleavage between the CAF and the PKD domain^31^, which gives rise to the N-terminal MαN fragment (harboring the NTF and the CAF) and the C-terminal MαC fragment (harboring the PKD domain and the RPT domain)^48^. Although the NTF is absent from the fibrils^33^, it is essential for their formation^44^. Its function is of a regulatory nature and appears to govern the activation and/or incorporation of the CAF into nascent fibrils. To achieve this, the NTF must be linked *in cis* to the CAF via an intact protein backbone^44^. This requirement distinguishes it from the accessory RPT domain, which can associate with the amyloid *in trans*^44^. A wildtype CAF subunit linked *in cis* to a mutant non-functional NTF (e.g. D73K) fails to be incorporated into nascent amyloid even in the presence of functional seeds^44^. Thus, the NTF is not merely required for amyloid seeding, but controls the insertion of new CAF subunits into growing fibrils.

How the NTF mechanistically activates the CAF or directs its incorporation into the fibrillar matrix is unknown, but its function has been mapped by alanine-scanning mutagenesis onto a 21 amino acid-long segment encompassing residues 70–90^44^. In the respective study, seven triplets of PMEL residues were exchanged against corresponding triplets of alanine (or sometimes glycine), which resulted in loss-of-function phenotypes in all cases except for mutant _85_SIA_87_ → AAG^44^. The remaining triplet mutants fell into two categories. The first category folded sufficiently well to pass ER quality control and accumulated in post-ER compartments without forming fibrils (_70_VSN_72_ → AAA, _73_DGP_75_ → AAA, _76_TLI_78_ → AAA, and _79_GAN_81_ → AGA)^44^. Of these triplet mutants, only one was further analyzed by substituting individual amino acids, which revealed that residues Asp-73 and Pro-75 are essential for PMEL amyloid formation^44^. The second category of mutants misfolded more severely, causing their full retention in the ER (_82_ASF_84_ → GAA and _88_LNF_90_ → AAA)^44^, where they only marginally interacted with conformation-sensitive antibodies^44^. Because these triplet mutants did not reach stage I melanosomes, the site of amyloid formation (Suppl. Fig. S1), it was impossible to determine whether the affected residues are relevant for CAF activation.

To address these issues and to identify functionally important residues in the NTF, we now targeted the respective region with milder alanine (or glycine) substitutions on the level of individual amino acids. This allowed all constructs to be released from the ER and to vigorously react with conformation-sensitive antibodies, indicating that misfolding was largely resolved or at least significantly reduced when compared to the previously generated set of triplet mutants. Our analysis based on well-established quantitative electron microscopy, immunofluorescence, and Western blotting assays identifies several critical residues within the NTF, some of which are absolutely essential for fibril formation.

## Results

### Folding, ER export, and early processing of NTF alanine-scanning mutants

Prior work has demonstrated that the 21 amino acid long regulatory NTF region within PMEL is structurally sensitive to mutations^44^. In order to reduce broad structural effects on PMEL folding as much as possible and to identify individual amino acids that are functionally critical for fibril formation, we constructed a set of point mutants, in which individual residues were substituted with alanine or glycine. Because Asp-73, Gly-74, and Pro-75 mutants have been described elsewhere^44^, we did not re-include them in this analysis. Hence, our set of mutants is comprised of 18 individual alanine- (or glycine-) scanning mutants.

**Figure 1:**
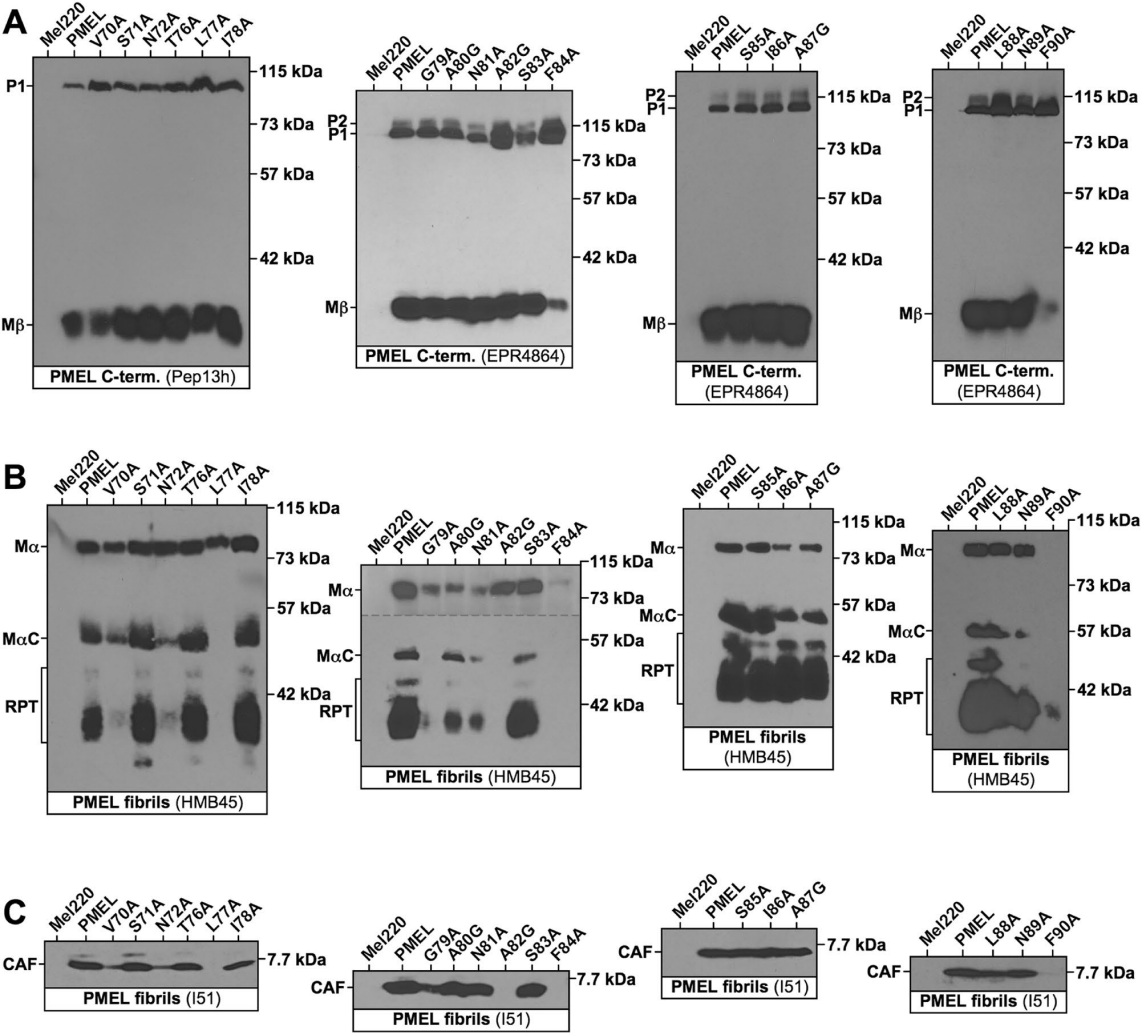
Proteolytic maturation of PMEL alanine-scanning mutants. Western blot analysis of SDS-lysed total membranes derived from Mel220 cells stably expressing PMEL alanine-scanning mutants. (**A**) PMEL-specific antibodies Pep13h and EPR4864 recognize the PMEL C-terminus. Note that P1 represents the ER form of the protein, while Mβ is generated through proteolytic cleavage in secretory compartments after PMEL has been released from the ER. The Mβ:P1 ratio is a measurement of the efficiency with which PMEL exits the ER, which past studies have shown is a good indicator for how well PMEL is folded. (**B**) Antibody HMB45 recognizes the sialylated fibril-associated fragments MαC and RPT. (**C**) Antibody I51 recognizes the fibril-forming CAF. Horizontal dashed lines separate different exposures of the same blot.

The constructs were stably expressed in the PMEL-deficient human melanoma cell line Mel220^49^. In this experimental system, we had previously determined that PMEL expression is somewhat higher (about two-fold) than endogenous PMEL levels in the human melanoma cell line buf1280^46^. We nevertheless note that even mild overexpression may to some extent affect PMEL trafficking, processing, and/or amyloidogenesis, which should be taken into account when interpreting the data. To characterize PMEL maturation, Western blots were employed using antibodies recognizing the C-terminus of the protein (Fig. 1A), the RPT domain (Fig. 1B), and the CAF (Fig. 1C). Selected relevant bands were quantified and are displayed in Suppl. Fig. S2. Because the PMEL C-terminus is not incorporated into melanosomal fibrils, Western blots targeting this domain visualize only newly synthesized, but not amyloid-incorporated protein. For all mutants, both the ER form (P1) as well as a post-ER form (Mβ) was detected (Fig. 1A). The observed Mβ:P1 ratio is considered a reasonable proxy for the ER export rate, and mutants prone to misfolding tend to show a drastic accumulation of P1 concomitant with a loss of significant levels of Mβ^46^. As shown in Fig. 1A, and in contrast to when this region was targeted with more aggressive triplet mutations^44^, none of the mutants was fully retained in the ER. In fact, only two mutants, F84A and F90A, showed a substantial reduction of Mβ levels and a concomitant increase in P1 levels, suggesting that the respective two aromatic amino acids could be moderately relevant for folding. Nevertheless, indicated by the presence of Mβ (Fig. 1A and Suppl. Fig. S2A), both F84A and F90A were exported from the ER to a significant extent. All other mutants behaved fairly normally with respect to their release from the ER.

In Mel220 cells, PMEL undergoes proteolytic cleavage into Mα and Mβ during secretion^23^. This cleavage occurred in all examined mutants as they gave rise to not only Mβ (Fig. 1A and Suppl. Fig. S2A), but also Mα (Fig. 1B). Mα levels were sharply reduced for F84A, however, while F90A-derived Mα was only visualizable on longer exposures than the one shown in Fig. 1B. This likely reflects the lower ER export efficiency of F84A and F90A, resulting in less PMEL substrate for proprotein convertases to act on. Altogether, we conclude that the majority of our NTF mutants undergoes largely normal secretory trafficking and early proteolytic maturation.

### NTF mutants traffic into melanosomes, but many fail to efficiently form fibrils and some show signs of PMEL misaggregation

**Figure 2:**
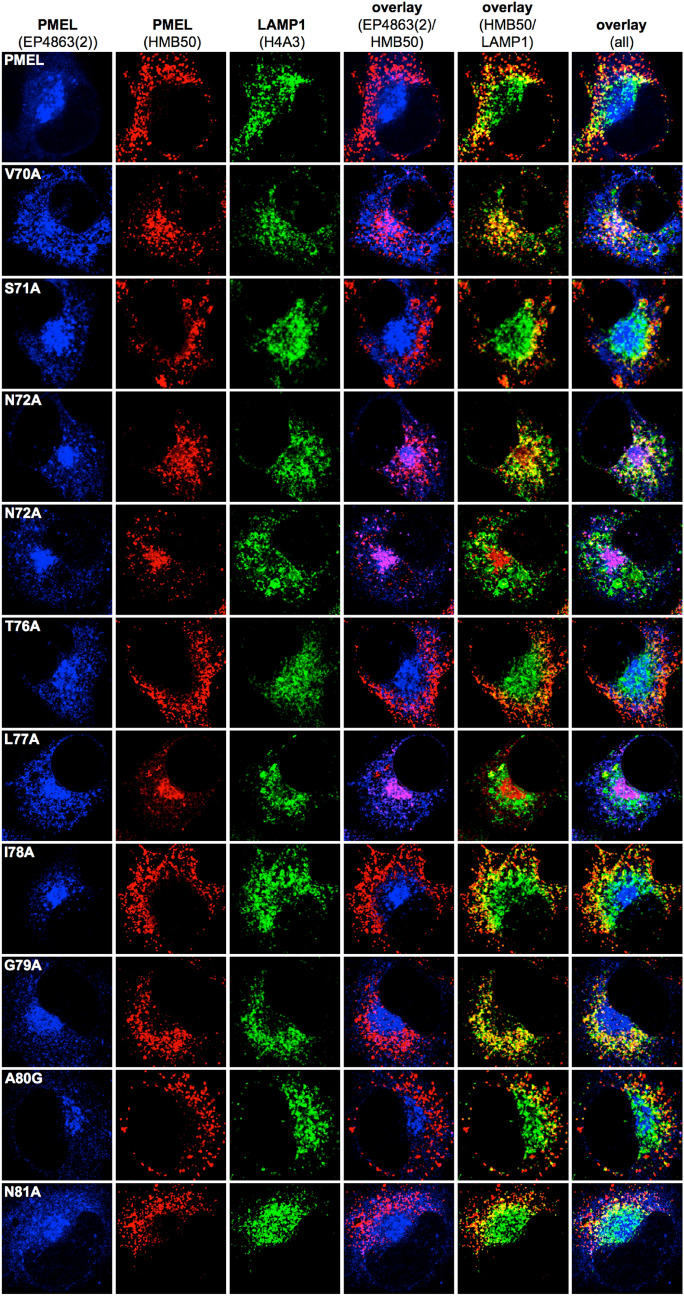
Subcellular trafficking and melanosome-lysosome segregation of PMEL alanine-scanning mutants. Immunofluorescence analysis of Mel220 cells stably expressing PMEL alanine-scanning mutants. The antibody EP4863(2) recognizes newly synthesized, but not fibrillar PMEL. The PMEL-specific antibody HMB50 recognizes mature melanosomal fibrils. The LAMP1-specific antibody H4A3 recognizes a lysosomal marker. Note that proper PMEL amyloid formation typically correlates in this assay with a significant separation of the perinuclear lysosomal staining (LAMP1) from the largely peripheral melanosomal staining (HMB50).

**Figure 3:**
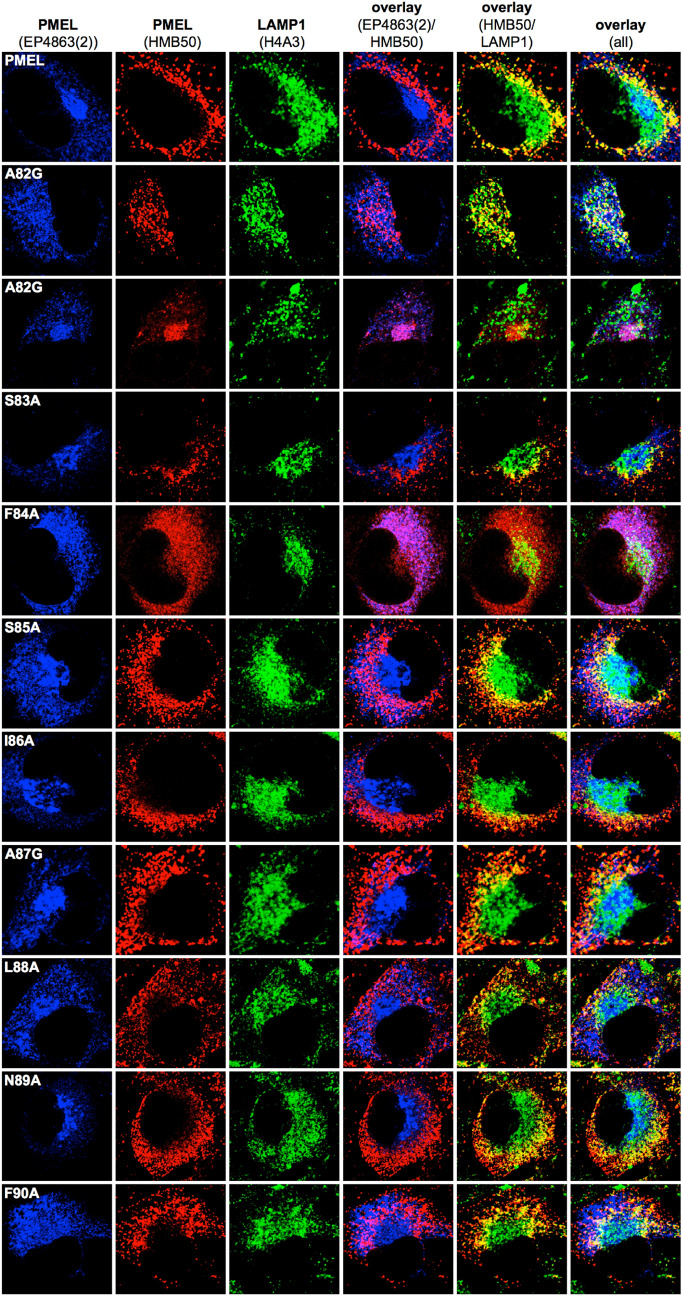
Subcellular trafficking and melanosome-lysosome segregation of PMEL alanine-scanning mutants. Immunofluorescence analysis of Mel220 cells stably expressing PMEL alanine-scanning mutants. The antibody EP4863(2) recognizes newly synthesized, but not fibrillar PMEL. The PMEL-specific antibody HMB50 recognizes mature melanosomal fibrils. The LAMP1-specific antibody H4A3 recognizes a lysosomal marker. Note that proper PMEL amyloid formation typically correlates in this assay with a significant separation of the perinuclear lysosomal staining (LAMP1) from the largely peripheral melanosomal staining (HMB50).

In order to monitor the subcellular distribution of newly synthesized PMEL, we performed immunofluorescence (IF) analysis using antibody EP4863(2), which recognizes the PMEL N-terminus^44^ (Figs. 2 and 3; *leftmost column*, and Suppl. Fig. S3A). In this assay, wildtype PMEL is found in the ER, the Golgi apparatus, and early stage melanosomes^44^ (Figs. 2 and 3; *topmost panel*). PMEL mutants mostly displayed EP4863(2) labeling consistent with this distribution (Figs. 2 and 3; *leftmost column*) indicating largely normal trafficking. Only mutants F84A and F90A were an exception, as they did not show significant levels of newly synthesized PMEL beyond the reticular pattern likely corresponding to the ER (Fig. 3; *rows 5 and 11*). This is in line with our Western blotting data (Fig. 1A) and probably reflects the low ER export of these two mutants.

Antibody HMB50 is conformation-sensitive and technically recognizes both newly synthesized as well as fibrillar PMEL^19^. However, likely because of the extremely high epitope density on PMEL fibrils, IF generally finds HMB50 labeling depleted from early secretory and stage I melanosomal compartments. Instead, practically all observable signal accumulates in compartments that contain mature fibrils, which in Mel220 cells are stage II melanosomes^19,44,46^. This is why the EP4863(2)-labeled and the HMB50-labeled populations of wildtype PMEL do not normally overlap in IF applications^19,44,46^ (Figs. 2 and 3; topmost row, column 4, and Suppl. Fig. S3A).

In fact, overlap of EP4863(2) labeling and HMB50 labeling in IF applications is a well-characterized signature of nonfunctional PMEL that fails to aggregate into amyloid form^46^. In the respective cells, epitope-rich fibrils which—under normal circumstances—would outcompete early compartments for HMB50 binding, are lacking and, hence, HMB50 antibody is free to bind to newly synthesized PMEL in the ER and Golgi among other organelles. Nearly complete overlap of EP4863(2) and HMB50 labeling by immunofluorescence was observed for PMEL mutants L77A (Fig. 2, *row 7, column 4*) and F84A (Fig. 3, *row 5, column 4*), indicating that Leu-77 and Phe-84 are essential for PMEL fibril formation. This overlap was also reflected in significantly higher Pearson’s coefficients for the EP4863(2) / HMB50 colocalization in the case of these mutants relative to wt-PMEL (Suppl. Fig. S3B). Interestingly, such overlap was not observed for mutant F90A, although this mutant was similarly impaired in ER export as mutant F84A (Fig. 1A). This suggests that beyond early PMEL folding in the ER, Phe-84 is additionally required for NTF function in melanosomes, while Phe-90 is not. Two further PMEL mutants, N72A and A82G, displayed overlap of EP4863(2) and HMB50 labeling in some (Fig. 2, *row 5, column 4*; Fig. 3, *row 3, column 4*) but not all cells (Fig. 2, *row 4, column 4*; Fig. 3, *row 2, column 4*). These results suggest that Asn-72 and Ala-82 are important, but not essential for NTF function. We note that heterogeneous phenotypes that are stronger in some cells and weaker in other cells of the same bulk transfectant cell line are frequently observed in our experimental system and have been described previously^44^.

In addition to EP4863(2) and HMB50, we also labeled the cells with the LAMP1-specific antibody H4A3. LAMP1 is present at high levels in lysosomes, but only at low levels in melanosomes^46^. We have described previously that in Mel220 cells, lysosomes concentrate in the perinuclear area, while melanosomes distribute into a peripheral horseshoe pattern. Thus, H4A3 labeling and HMB50 labeling are typically segregated in wildtype PMEL-expressing cells (Figs. 2 and 3; *topmost row, column 5*). However, in previous studies we have reported many PMEL mutants displaying aberrant misaggregation of PMEL. A shared signature of these mutants is the sometimes partial and sometimes complete accumulation of PMEL aggregates in lysosomes instead of melanosomes^31,44,46^. In the respective cases, full overlap of H4A3 and HMB50 labeling is seen in the perinuclear region^46^. Interestingly, this signature of misaggregation was strongly observed for mutants V70A and G79A (Fig. 2, *rows 2 and 9, column 5*), indicating that Val-70 and Gly-79 are required for proper PMEL amyloid formation. In addition, N72A and A82G cells that did not display the “lack of aggregation phenotype” as discussed above, invariably showed the misaggregation signature (Fig. 2, *row 4, column 5*; Fig. 3, *row 2, column 5*). Thus, it seems that mutating Asn-72 and Ala-82 not only strongly reduces PMEL fibril formation overall, but that in those cells in which aggregation does occur at a low level, the process is abnormal and aggregates are largely deposited in lysosomal LAMP1^high^ compartments. Loss-of-function mutants L77A and F84A displayed reduced colocalization with LAMP1, likely reflecting their lack of accumulation in either melanosomes or lysosomes (Suppl. Fig. S3C).

The phenotypes observed by IF were recapitulated in our Western blotting results with antibodies HMB45 (Fig. 1B) and I51 (Fig. 1C). Both these antibodies react with PMEL regions that are associated with fibrils, such as the RPT domain (HMB45) and the CAF (I51). We have shown previously that the CAF is extremely unstable and only accumulates to levels detectable by Western blotting, if it is stabilized by aggregation (i.e. incorporated into melanosomal amyloid fibrils)^44^. Accordingly, no CAF was detected for loss-of-function mutants L77A and F84A (Fig. 1C). Similar to the CAF, MαC is also unstable, if it is not incorporated into fibrils. Hence, in the absence of fibril formation, MαC fails to accumulate to detectable levels, while downstream processing of MαC into the characteristic RPT fragments depends on active fibril formation in the cell^44^. Accordingly, antibody HMB45 detected only the Mα precursor, but neither MαC nor RPT fragments in lysates of mutants L77A and F84A (Fig. 1B). This further supports the view that these are loss-of-function mutants.

Misaggregation, in our experience, also frequently leads to reduced detection of fibrillar material by Western blotting^46^, probably because misaggregated amyloid is not readily solubilized by boiling in 1% SDS and large aggregates fail to enter SDS-PAGE gels. This likely explains the drastic reduction of fibrillar material observed with both the HMB45 and the I51 antibody for mutants V70A, N72A, G79A, and A82G (Fig. 1B/C), which are the mutants displaying the misaggregation signature by IF (Figs. 2 and 3).

**Figure 4:**
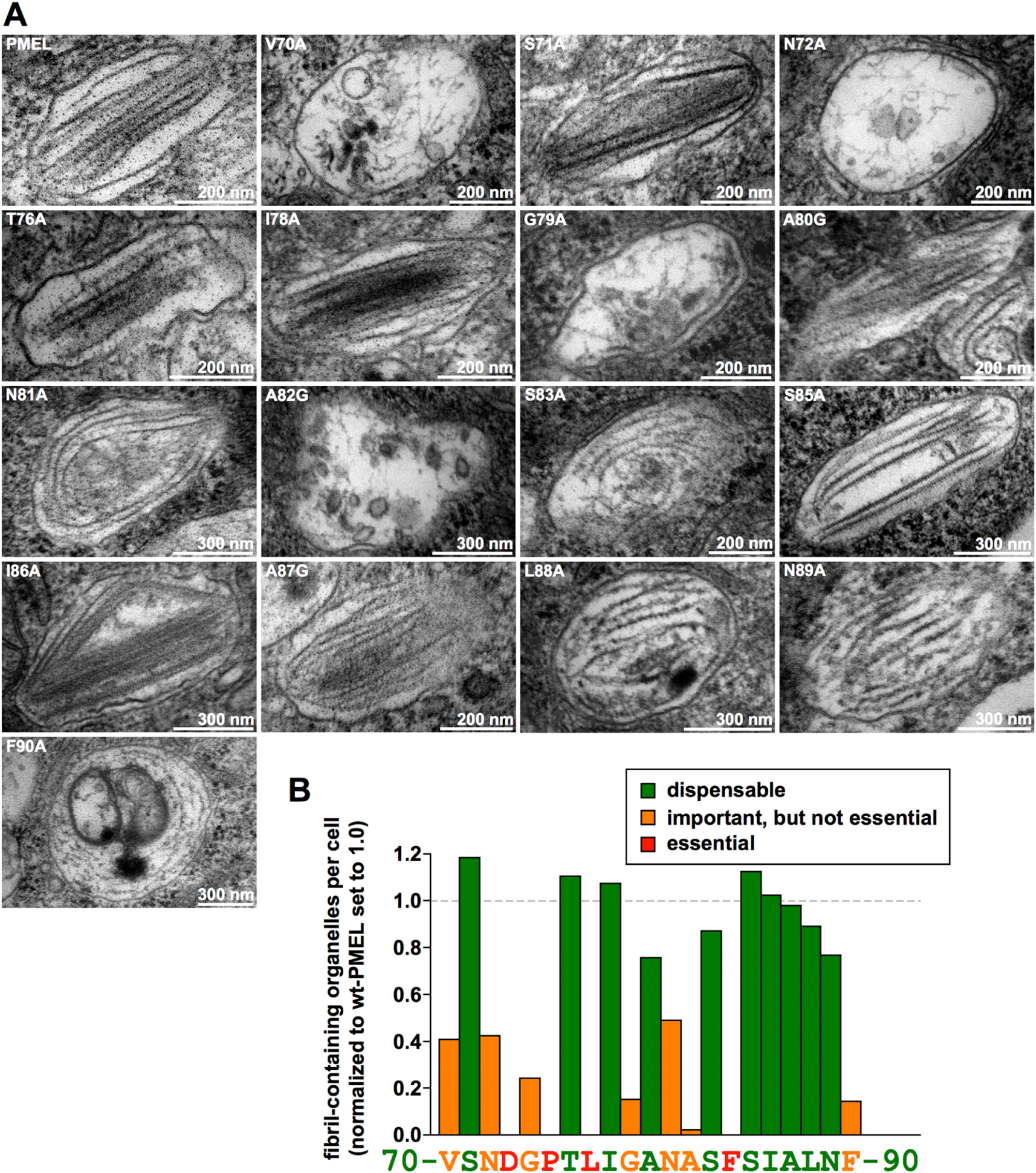
Melanosomal amyloid formation by PMEL alanine-scanning mutants. (**A**) EM analysis of Epon-embedded stable Mel220 transfectants expressing PMEL alanine-scanning mutants. Melanosomes of mutants that form fibrils are shown. The respective quantification of fibril formation in the indicated cell lines is depicted in Suppl. Fig. S4 B–E and summarized in (**B**). (**B**) Quantitative EM analysis of PMEL alanine-scanning mutants. Shown is the number of fibril-containing organelles per cell [N = 15] after normalization to wt-PMEL (set to 1)). Essential (category 3), relevant (category 2), and largely dispensable residues (category 1) are colored in red, orange, and green, respectively.

Finally, we directly analyzed fibril formation of our mutants using electron microscopy (EM) (Fig. 4A). The quantification of the EM results is summarized in Fig. 4B and shown in a more detailed fashion including a statistical analysis in Suppl. Fig. S4B–E. For the purpose of providing context and to show the complete 21 amino acid-long motif, Fig. 4B also includes the quantification from the published data for mutants D73A, G74A, P75A^44^. Consistent with our Western blotting (Fig. 1B/C) and IF results (Figs. 2 and 3), mutants L77A and F84A, were shown to lack fibrils (Fig. 4B and Suppl. Fig. S4A). This confirms in a third independent assay that residues Leu-77 and Phe-84 are essential for fibril formation. Additionally, mutants V70A, N72A, G79A, and A82G not only showed dramatically reduced fibril formation overall (Fig. 4B), but also very low fibril load per organelle. In these mutants, amyloid was mostly observed in immature-appearing melanosomes and the hair-thin fibrils that were seen resembled unassembled and largely disorganized protofibrils rather than mature fibrillar amyloid (Fig. 4A).

## Discussion

The formation of amyloid was long regarded as a strictly pathological process. Amyloid fibrils and prefibrillar oligomers are well-known to damage membranes, to be toxic to cells, and to either directly cause or at least to contribute to neurodegenerative diseases including Alzheimer’s, Parkinson’s and Huntington’s disease as well as to various other amyloidoses^50–54^. The discovery that amyloids can also represent physiological structures that serve crucial cellular functions surprised many in the field, and an increasing number of examples has been found in recent years^55–58^. PMEL is one of these functional amyloids, playing a key role in vertebrate pigmentation^2^.

It has been noted that amyloid formation is often efficient in acidic organelles, such as lysosomes or early melanosomes^52,59–61^, which is likely not a coincidence. Changes in pH can drive conformational rearrangements favoring aggregation, for instance by local structural unfolding leading to the exposure of new interaction interfaces that were previously buried in the protein core^62^. It is therefore not surprising that low pH as found in endosomes and lysosomes has been reported to support amyloid formation in both physiological as well as pathological systems^62–70^. In pH-sensitive proteins, the pK_a_ value of the imidazole side chain of histidine, which is close to 6.1, is frequently exploited to drive pH-responsive conformational rearrangements^71–73^. The pK_a_ value of the negatively charged carboxylate group of aspartic and glutamic acid is typically lower (~ 3.9 and ~ 4.3, respectively)^74^, but can shift significantly depending on the molecular environment in which these amino acids are located within a protein^75^. Hence, aspartic and glutamic acid residues also frequently act as pH sensors responding to moderately acidic conditions^74,76–79^.

**Figure 5:**
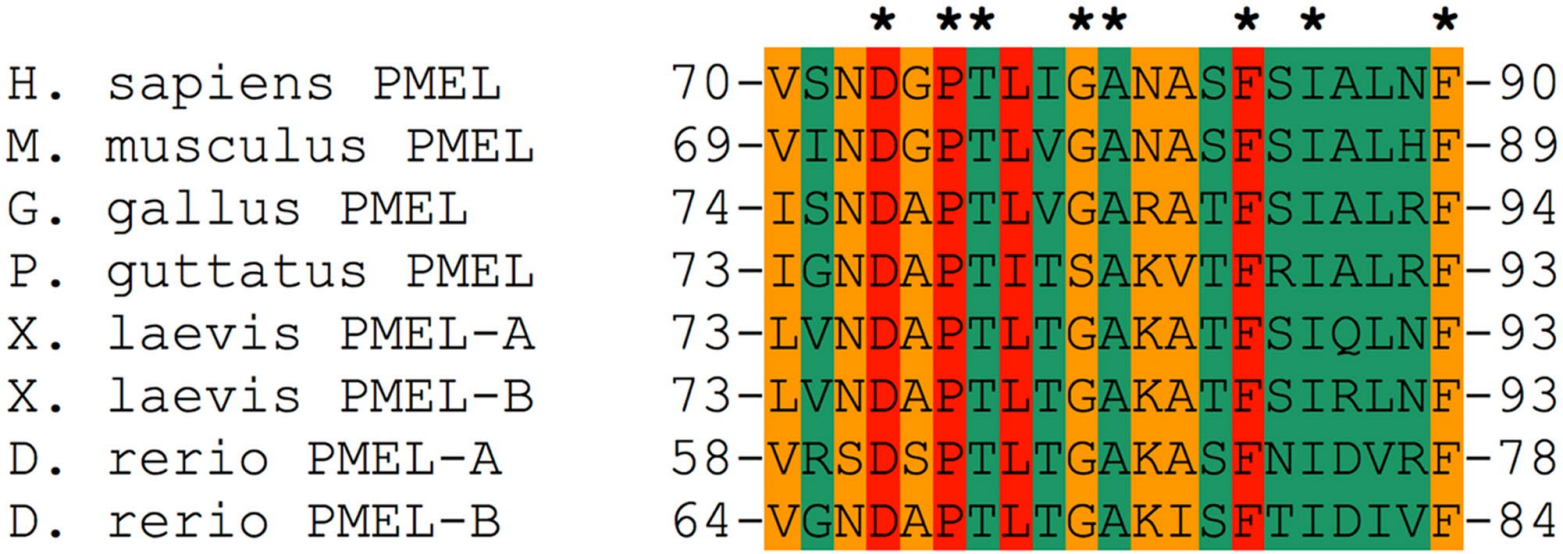
Evolutionary conservation of the 21 amino acid regulatory motif in the PMEL N-terminus. Evolutionary conservation of PMEL residues 70–90 in mammals, birds, reptiles, amphibians, and fish. The coloring scheme is taken from Fig. 4B (essential residues, red; important, but not essential residues, orange; largely dispensable residues, green). Residues that are 100% conserved are labeled with an asterisk.

In this context, we note that the regulatory motif in the PMEL NTF contains a prominent aspartate residue, Asp-73, which is both absolutely essential for melanosomal amyloid formation and completely conserved in evolution^44^ (Figs. 4B and 5). We propose that Asp-73 could act as a pH sensor responding to the acidic conditions found within early stage melanosomes^26^. Such a scenario would explain why PMEL selectively aggregates within pigmentation-associated organelles, while premature and potentially toxic aggregation along the secretory route is not observed. Indeed, a related strategy is employed by spidroins, the proteins forming spider silk, which is an amyloid-like substance^58^. Specifically, spidroins have a similar domain architecture as PMEL, in that a regulatory N-terminus is adjacent to a central aggregation-prone region^80^. In the silk gland of the spider, spidroins are stored at very high concentrations in a soluble form at neutral pH^65^. However, after their expulsion into the spinning duct, where pH sharply drops, a cluster of glutamic acid residues undergoes rapid protonation^65^. This leads to structural changes and dimer formation, which in turn is essential for the activation of amyloid aggregation^65,81,82^. Hinting at a generalizable function, the spidroin N-terminus cannot only suppress the aggregation of spider silk, but when fused to other amyloidogenic proteins including human Aβ, can also confer solubility on these^83,84^.

Potential conformational changes driven by Asp-73 protonation could be coordinated either upstream or downstream with disulfide bond rearrangements within PMEL, which others have suggested to precede fibril formation^24^. Moreover, the specific lipid context and/or high curvature of ILVs could create an environment further supporting structural alterations and partial PMEL unfolding, allowing for the exposure of the amyloidogenic CAF. Residues characterized as critical in this study could be involved in these steps. Indeed, lipid composition and curvature of vesicles can have a profound effect on amyloid formation in other systems^85,86^. Thus, it is plausible to speculate that it may be a cascade of possibly interdependent sequential activation steps that impose a tight control on PMEL aggregation. The regulatory motif in the NTF could be at the heart of integrating environmental signals and translating them into a mechanism driving the activation and priming of CAF subunits for their controlled incorporation into nascent fibrils.

One notable observation is that mutations V70A, N72A, G79A, and A82G appear to negatively affect amyloid assembly not merely suppress amyloid formation (i.e. we did not simply observe a reduced load of morphologically normal amyloid sheets in melanosomes, but instead found aberrant, disorganized and often isolated fibrils) (Fig. 4A). Underlying this phenotype could be a defect of the respective mutant fibrils to associate laterally into the characteristic amyloid sheets^36,45^. Interestingly, this resembles observations with PMEL mutant R191S, which we had reported previously^46^. However, unlike Arg-191, which is a residue inside the CAF, Val-70, Asn-72, Gly-79, and Ala-82 are residues located in the NTF, and the PMEL N-terminus does not accumulate in the fibrils^44^. Thus, if these residues control lateral fibril assembly in a direct manner, they would probably exert this function during a hypothetical transient and short-lived association of the NTF with the nascent amyloid, during incorporation of a new MαN subunit, and before the PMEL N-terminus is proteolytically released. Alternatively, the four amino acids could affect lateral fibril assembly in an indirect manner through controlling local folding at the NTF-CAF interface and/or by guiding structural rearrangements that perhaps position Arg-191 optimally to later mediate lateral fibril incorporation into the growing sheets.

Ultimately, unassembled isolated fibrils in V70A-, N72A-, G79A-, or A82G-expressing cells could face one of two fates. Either they could undergo rapid degradation, which could explain why we observe a pronounced “lack of aggregation signature” in our IF experiments for mutants N72A and A82G (Fig. 2, *row 5*, and Fig. 3, *row 3*, and Suppl. Fig. S3B). As discussed above, the underlying reason for this signature is the freeing up of fibril-reactive HMB50 antibody to bind to earlier forms of the PMEL protein present in secretory compartments as highly epitope-dense aggregates fail to accumulate to levels at which they would effectively outcompete these forms for antibody binding. A second, alternative path that unassembled fibrils could take is to structurally collapse into malformed aggregates, which would subsequently build up in lysosomes. This would be consistent with the at least partial “misaggregation signature” that mutants V70A, N72A, G79A, and A82G display (Fig. 2, *rows 2, 4 and 9*, and Fig. 3, *row 2*). In different cells of the same mutant cell line either the degradation pathway or the misaggregation pathway might dominate, depending on how rapidly fibrils can be cleared, which could explain some of the heterogeneity we observe in our immunofluorescence samples (Figs. 2 and 3).

However, it is not unlikely that several or all of these mutations are also slowing or otherwise reducing the formation of melanosomal fibrils rather than merely impairing their higher order assembly. In such a scenario, they would represent milder versions of the full loss-of-function mutants L77A and F84A (Fig. 4B). In line with this, PMEL triplet mutations _70_VSN_72_ → AAA and _79_GAN_81_ → AGA both cause complete loss-of-function phenotypes^44^ very similar to single amino acid mutations D73A^44^ or L77A (Fig. 4B). These triplet mutations combine mutations V70A and N72A, or G79A and N81A, respectively, which are in the “important, but not essential category” (Fig. 4B, *shown in orange*). Without convincing support from structural data it is hard to provide a strong hypothesis on the role that these residues and also the essential residues Leu-77 and Phe-84 are playing mechanistically during PMEL fibril formation. Nevertheless, one possibility is that they could be involved in releasing the CAF from an inhibitory interaction in response to Asp-73 protonation. This could, for instance, occur by breaking a hypothetical NTF-CAF domain interface, which one could assume is keeping the CAF in an inactive state until the final cascade of maturation and processing begins (Suppl. Fig. S1). Alternatively, one could speculate that these residues are structurally involved in the formation of the biochemical environment around Asp-73, fine-tuning the pK_a_ of its carboxylate group in order to allow efficient protonation under physiological conditions in early-stage melanosomes.

Finally, functionally relevant residues in the regulatory NTF motif could be involved in critical interactions with factors that are more generally involved in PMEL amyloidogenesis. One of these factors is ApoE, which was recently speculated to serve as part of a nucleation and assembly platform promoting PMEL aggregation on ILVs within early melanosomes^87^. In this context it is interesting to note that an siRNA-mediated knockdown of ApoE was reported to result in the formation of unstructured aggregates in roundish rather than ellipsoid melanosomes^87^, perhaps not unsimilar to our observations with PMEL mutants V70A, N72A, G79A, and A82G. Thus, we consider the possibility that these residues could functionally interact with ApoE during fibril formation. Should such interactions be relevant for amyloid nucleation on the ILV surface, this could also provide an alternative explanation for the reduced fibril load observed in the context of the respective mutations (Fig. 4A/B).

The importance of the regulatory motif in the NTF is also supported by the high degree of conservation, particularly of the functionally most important residues (Figs. 4 and 5). Future structural studies may benefit from our detailed characterization of these amino acids, which would be necessary to better understand the molecular mechanism by which they are controlling amyloid formation. Interestingly, when a recent study identified a number of PMEL mutations associated with human eye disease^13^, none of these mutations were located in the NTF regulatory region, while only one mutation was found in the CAF. In fact, most mutations seemed to concentrate within the accessory RPT domain and none appeared to fully disrupt fibril formation^13^. Mutations in the regulatory NTF motif as well as in the CAF frequently have drastic effects, often completely abrogating amyloid formation^31,44^. Thus, the collection of disease-associated PMEL mutations might hint at gain-of-function mutations, potentially driving toxic misaggregation, to have stronger phenotypic effects than true loss-of-function mutations. Indeed, this is consistent with previous observations^14^ and could have major implications for understanding the mechanism underlying disease progression.

## Material and methods

### Cell lines and cell culture

LG2-MEL-220 (Mel220) is a human PMEL-deficient melanoma cell line^49^ and was grown in IMDM (Sigma)/10% FCS (HyClone) containing non-essential amino acids (Gibco), GlutaMax (Gibco), and penicillin/streptomycin (Gibco). PMEL transfectants were cultured in medium additionally containing 2 mg/ml G418 (Gibco). PMEL expression, subcellular localization, and reactivity with various specific antibodies in Mel220 cells has been extensively characterized in previous work^23,46^.

### Vector constructs and PMEL expression

Human PMEL mutants were cloned using a standard QuikChange mutagenesis approach^88^ employing human PMEL in pBMN-IRES-neo^46^ as a template in combination with primer pairs listed in Suppl. Table S1.

All vectors were sequenced before retroviral transduction^44^ into Mel220 cells and selection with 2 mg/ml G418 (Gibco).

### Antibodies

I51 is a rabbit polyclonal antiserum raised against residues 206–220 in the PMEL CAF^33^. Pep13h is a rabbit polyclonal antiserum raised against the carboxyterminal 15 residues of PMEL^59^. HMB50 is a conformation-sensitive mouse monoclonal antibody recognizing an epitope in PMEL that is contained within residues 224–254, likely located in the PKD domain^19,29,31^. HMB45 (NeoMarkers) is a mouse monoclonal antibody recognizing residues 328–344 in the sialylated RPT domain^34,48^. EP4863(2) (Abcam) and EPR4864 (Abcam) are rabbit monoclonal antibodies recognizing the PMEL N-terminus (NTF)^44^ and the PMEL C-terminus^36^, respectively. We had previously mapped the EP4863(2) epitope to PMEL residues 28–75^31^. H4A3 (Abcam) is a mouse monoclonal antibody recognizing LAMP1. HRP- and fluorophore-labeled secondary antibodies were purchased from Jackson ImmunoResearch and Molecular Probes.

### Western blotting and immunofluorescence

Total membranes were prepared as described^89^. Briefly, we resuspended 5 × 10^6^ cells in 1 ml ice-cold 10 mM Tris–HCl pH 7.4 containing protease inhibitor (Complete, Roche) and incubated on ice for 10 min. Lysed cells were Dounce-homogenized with 30 strokes using the “tight” pestle and centrifuged at 800*g* and 4 °C for 10 min. The resulting supernatant was spun at 45,000 rpm and 4 °C using the TLA-55 rotor in a Beckman Optima TL ultracentrifuge. The pellet was subsequently lysed in PBS/1% SDS/1% β-mercaptoethanol for 10 min at RT followed by 10 min at 95 °C and subjected to SDS-PAGE. Western blotting was carried out as described^90^. For compliance with journal guidelines, extended images of the films corresponding to the experiments shown in Fig. 1 are provided in Suppl. Fig. S5.

Immunofluorescence was performed as described^91^. Briefly, we fixed and permeabilized Mel220 transfectants before staining with primary antibodies in PBS/0.5% BSA/0.5% saponin for 1 h followed by staining with Alexa647-, Alexa546-, or Alexa488-conjugated secondary antibodies in the same buffer at 1:100. Primary antibodies H4A3 (0.100 mg/ml), EP4863(2) (0.357 mg/ml), and HMB50 (hybridoma supernatant) were used at 1:25, 1:100, and 1:100, respectively. Cells were mounted in ProLong Gold reagent (Invitrogen). Images were acquired using a Leica TCS SP8 confocal microscope (Leica Microsystems) at 63 × magnification.

### Electron microscopy

For Epon embedding, we fixed the cells in 2.5% glutaraldehyde / 2% sucrose in 0.1 M sodium cacodylate buffer pH 7.4 (NaCaCo buffer) for 30 min at RT followed by 30 min at 4 °C. Subsequently, cells were rinsed with NaCaCo buffer and further processed as described^46^. Samples were viewed using an FEI Tecnai Biotwin TEM at 80 kV. Images were collected using Morada CCD and iTEM (Olympus) software.

Epon-embedded EM samples were first inspected to qualitatively assess if cells formed fibril-containing melanosomes. To quantify fibril formation, we then counted in one view field fibril-containing organelles in 15 arbitrarily chosen cells. Each count was performed once and the respective means are shown in Suppl. Fig. S4B–E. A One-way ANOVA test followed by Dunnett's post-test was used to determine whether the mean is statistically different from the wt-PMEL sample. Asterisks in respective figures indicate statistical significance (*p < 0.05; **p < 0.01; ***p < 0.001; NS, not significant).

## Supplementary Information

Supplementary Information

## Data Availability

The datasets generated during and/or analyzed during the current study are available from the corresponding author on reasonable request.
