# Supplementary material for: Identification of critical amino acid residues in the regulatory N-terminal domain of PMEL
Source: Sci Rep. 2021 Apr 8;11:7730. doi: 10.1038/s41598-021-87259-y (PMC8032716; doi:10.1038/s41598-021-87259-y)
Supplement: Supplementary file 1 — Supplementary Information [file 41598_2021_87259_MOESM1_ESM.pdf]

Supplementary Material

Identification of critical amino acid residues in the regulatory N-terminal domain of PMEL

Susan M. Mitchell[a,1], **Morven Graham**[b,1], **Xinran Liu**[b], **and Ralf M. Leonhardt**[a,*]

From the [a] Department of Immunobiology and the [b] Department of Cell Biology, Yale University School of Medicine, 300 Cedar Street, New Haven CT 06519, USA

[1] equal contribution

[*] Address correspondence to: Ralf M. Leonhardt, Ph.D., Boehringer Ingelheim RCV GmbH & Co KG, Cancer Immunology & Immune Modulation, Dr. Boehringer Gasse 5-11, A-1121 Vienna, Austria,

Tel. +43 1 80105-9310;

E-mail: Ralf.Leonhardt@boehringer-ingelheim.com

SUPPLEMENTARY TABLE LEGEND

Suppl. Table S1. Primers used for QuikChange mutagenesis of PMEL. The indicated primers were used to introduce alanine/glycine mutations into PMEL in expression vector pBMN-IRES-neo.

SUPPLEMENTARY FIGURE LEGENDS

Suppl. Figure S1. Domain architecture and processing pathway of PMEL. (A) The PMEL Mα fragment harbors several prominent lumenal domains: The N-terminal fragment (NTF) (*yellow*), the core amyloid fragment (CAF) (*blue*), the polycystic kidney disease (PKD)-like domain (*grey*), and the repeat (RPT) domain (*red*). (B) PMEL is synthesized into the ER membrane as the so-called P1 form. In the Golgi, the protein undergoes extensive O-glycosylation and maturation of N-linked glycans, which gives rise to the P2 form. P2 is cleaved by a protease of the proprotein convertase family, likely furin, which results in a membrane-standing Mβ fragment and a lumenal Mα fragment. Mα and Mβ remain linked to each other via a disulfide bond. After leaving the trans-Golgi network (TGN), PMEL migrates further to the plasma membrane and, subsequently, undergoes endocytosis and traffics into an early melanosomal compartment. There, the β-secretase BACE-2 cleaves a soluble Mα fragment off the membrane. Next, Mα undergoes a further cleavage between the CAF and the PKD domain, resulting in an N-terminal MαN and a C-terminal MαC fragment. Either immediately before, during, or immediately after the incorporation of the CAF in to the nascent core amyloid, the NTF is cleaved away. Finally, MαC is embedded into the core amyloid, which is formed by the CAF. This last step serves to promote the characteristic spacing of the PMEL amyloid fibrillar sheets, which is mediated via the extensive O-glycosylation of the RPT domain. Further processing of MαC within the fibrils results in the RPT fragments reactive with antibody HMB45.

Suppl. Figure S2. Quantification of Western blots showing PMEL alanine-scanning mutants. (A-C) Selected PMEL forms, such as Mβ (A), RPT (B), and the CAF (C) shown in the Western blots of Figure 1A, 1B, and 1C, respectively, were quantified using ImageJ. Results are shown as bar diagrams, in which the corresponding wildtype PMEL form is set to 100%.

Suppl. Figure S3. Characterization of the immunofluorescence colocalization pattern of PMEL alanine-scanning mutants. (A) Mel220 cells transduced with PMEL (*bottom row*) or parental Mel220 cells not expressing PMEL (*top row*) were labeled with antibodies recognizing newly synthesized PMEL (EP4863(2)) (*blue*), fibrillar PMEL (HMB50) (*red*), or LAMP1 (H4A3) (*green*). (B) Colocalization of HMB50 and EP4863(2) labeling and (C) colocalization of HMB50 and LAMP1 labeling in the indicated Mel220 transfectants was assessed using the JACoP plugin for ImageJ. Average Pearson's coefficients determined in five representative images of each cell line are depicted as a bar diagram. Error bars show the standard deviation from the mean. The coloring scheme follows the pattern employed in Figure 4 highlighting essential residues (*red*), important, but not essential residues (*orange*), and dispensable residues (*green*). Asterisks indicate the result of a One-way ANOVA test followed by Dunnett's post-test using wildtype PMEL as control column (*, $p < 0.05$; ***, $p < 0.001$; ****, $p < 0.0001$).

Suppl. Figure S4. Fibril formation by PMEL alanine-scanning mutants. (A) Representative EM images of a fibril-containing melanosome in PMEL-expressing Mel220 cells (*left panel*) and of fibril-free endocytic organelles in Mel220 cells expressing PMEL loss-of-function mutants L77A and F84A (*middle and right panel*). (B-E) EM analysis of Mel220 transfectants showing the number of fibril-containing organelles per cell [N=15]. Asterisks indicate the result of a One-way ANOVA test followed by Dunnett's post-test using wildtype PMEL as control column (**, $p < 0.01$; ***, $p < 0.001$; NS, not significant).

Suppl. Figure S5. Additional extended image versions of Western blotting experiments from Fig. 1. (A-C) For compliance with journal guidelines, we are providing supplementary image versions of the Western blotting experiments in Fig. 1A, B, and C, showing extended regions of the respective membranes beyond the relevant area of interest. As a consequence, neighboring bands on the same blot that belong to different, unrelated experiments are occasionally seen (*e.g.*, lane 1 in the leftmost panel of Suppl. Fig. 5A or the last lane in the leftmost panel of Suppl. Fig. 5B). After blotting, membranes were usually cut into individual pieces to be stained separately with the indicated antibodies. For staining of the small CAF

fragment the lower part of a membrane was regularly used (cut between the 22 kDa the 16 kDa marker band), while the corresponding upper part was most frequently stained using antibodies against the PMEL C-terminus (*e.g.*, Pep13h or EPR4864) or the PMEL RPT domain (HMB45). Following the staining, membrane pieces were assembled back in their original positions before exposing a film. Red brackets highlight the region stained with the respective antibody of interest (*e.g.*, in Suppl. Fig. S5C this is the staining with I51-specific antibody recognizing the CAF), whereas regions outside the red brackets and bands therein correspond to other parts of the membrane stained with a different antibody. One example for this are the rightmost panels in Suppl. Fig. 5A and 5C, respectively, which both show the same Mβ bands, because in this experiment the upper part of the membrane was stained with antibody EPR4864 (recognizing Mβ), while the lower part of the membrane was stained with antibody I51 (recognizing the CAF. Unlike in Fig. 1C, however, the lower part is now no longer cropped to the (I51-stained) area of interest, but instead shows part of the adjacent EPR4864-stained area which is on the same film. Borders of the individual membrane pieces are almost never visible, except on very long exposures as in Suppl. 5B, second bottom panel. The order of the images corresponds to the order of the images in Fig. 1. (A) PMEL C-terminus. (B) PMEL RPT domain. (C) PMEL CAF. The top and bottom panel in the second column of Suppl. Fig. S5 corresponds to the short and the long exposure of the same membrane, which is displayed as a composite image in Fig. 1B.

Supplementary Table S1, ***Primers used for QuikChange mutagenesis of PMEL.***

V70A	5′-GTGTCCCTCAAGGCCAGTAATGATGGG-3′ *(forward)*<br>5′-CCCATCATTACTGGCCTTGAGGGACAC-3′ *(reverse)*
S71A	5′-GTCCCTCAAGGTCGCTAATGATGGGCCTAC-3′ *(forward)*<br>5′-GTAGGCCCATCATTAGCGACCTTGAGGGAC-3′ *(reverse)*
N72A	5′-CCTCAAGGTCAGTGCTGATGGGCCTACAC-3′ *(forward)*<br>5′-GTGTAGGCCCATCAGCACTGACCTTGAGG-3′ *(reverse)*
T76A	5′-GTAATGATGGGCCTGCACTGATTGGTGC-3′ *(forward)*<br>5′-GCACCAATCAGTGCAGGCCCATCATTAC-3′ *(reverse)*
L77A	5′-GTAATGATGGGCCTACAGCGATTGGTGCAAATGC-3′ *(forward)*<br>5′-GCATTTGCACCAATCGCTGTAGGCCCATCATTAC-3′ *(reverse)*
I78A	5′-GATGGGCCTACACTGGCTGGTGCAAATGCC-3′ *(forward)*<br>5′-GGCATTTGCACCAGCCAGTGTAGGCCCATC-3′ *(reverse)*
G79A	5′-CCTACACTGATTGCTGCAAATGCCTCC-3′ *(forward)*<br>5′-GGAGGCATTTGCAGCAATCAGTGTAGG-3′ *(reverse)*
A80G	5′-CTACACTGATTGGTGGAAATGCCTCCTTC-3′ *(forward)*<br>5′-GAAGGAGGCATTTCCACCAATCAGTGTAG-3′ *(reverse)*
N81A	5′-CACTGATTGGTGCAGCTGCCTCCTTCTCT-3′ *(forward)*<br>5′-AGAGAAGGAGGCAGCTGCACCAATCAGTG-3′ *(reverse)*
A82G	5′-GATTGGTGCAAATGGCTCCTTCTCTATTGC-3′ *(forward)*<br>5′-GCAATAGAGAAGGAGCCATTTGCACCAATC-3′ *(reverse)*
S83A	5′-TTGGTGCAAATGCCGCCTTCTCTATTGC-3′ *(forward)*<br>5′-GCAATAGAGAAGGCGGCATTTGCACCAA-3′ *(reverse)*
F84A	5′-GGTGCAAATGCCTCCGCCTCTATTGCATTG-3′ *(forward)*<br>5′-CAATGCAATAGAGGCGGAGGCATTTGCACC-3′ *(reverse)*
S85A	5′-GCAAATGCCTCCTTCGCTATTGCATTGAAC-3′ *(forward)*<br>5′-GTTCAATGCAATAGCGAAGGAGGCATTTGC-3′ *(reverse)*
I86A	5′-TGCCTCCTTCTCTGCTGCATTGAACTTCC-3′ *(forward)*<br>5′-GGAAGTTCAATGCAGCAGAGAAGGAGGCA-3′ *(reverse)*
A87G	5′-CCTCCTTCTCTATTGGATTGAACTTCCCTGG-3′ *(forward)*<br>5′-CCAGGGAAGTTCAATCCAATAGAGAAGGAGG-3′ *(reverse)*
L88A	5′-CCTTCTCTATTGCAGCGAACTTCCCTGGAAGC-3′ *(forward)*<br>5′-GCTTCCAGGGAAGTTCGCTGCAATAGAGAAGG-3′ *(reverse)*
N89A	5′-CTCTATTGCATTGGCCTTCCCTGGAAGC-3′ *(forward)*<br>5′-GCTTCCAGGGAAGGCCAATGCAATAGAG-3′ *(reverse)*
F90A	5′-CTATTGCATTGAACGCCCCTGGAAGCCAAAAGG-3′ *(forward)*<br>5′-CCTTTTGGCTTCCAGGGGCGTTCAATGCAATAG-3′ *(reverse)*

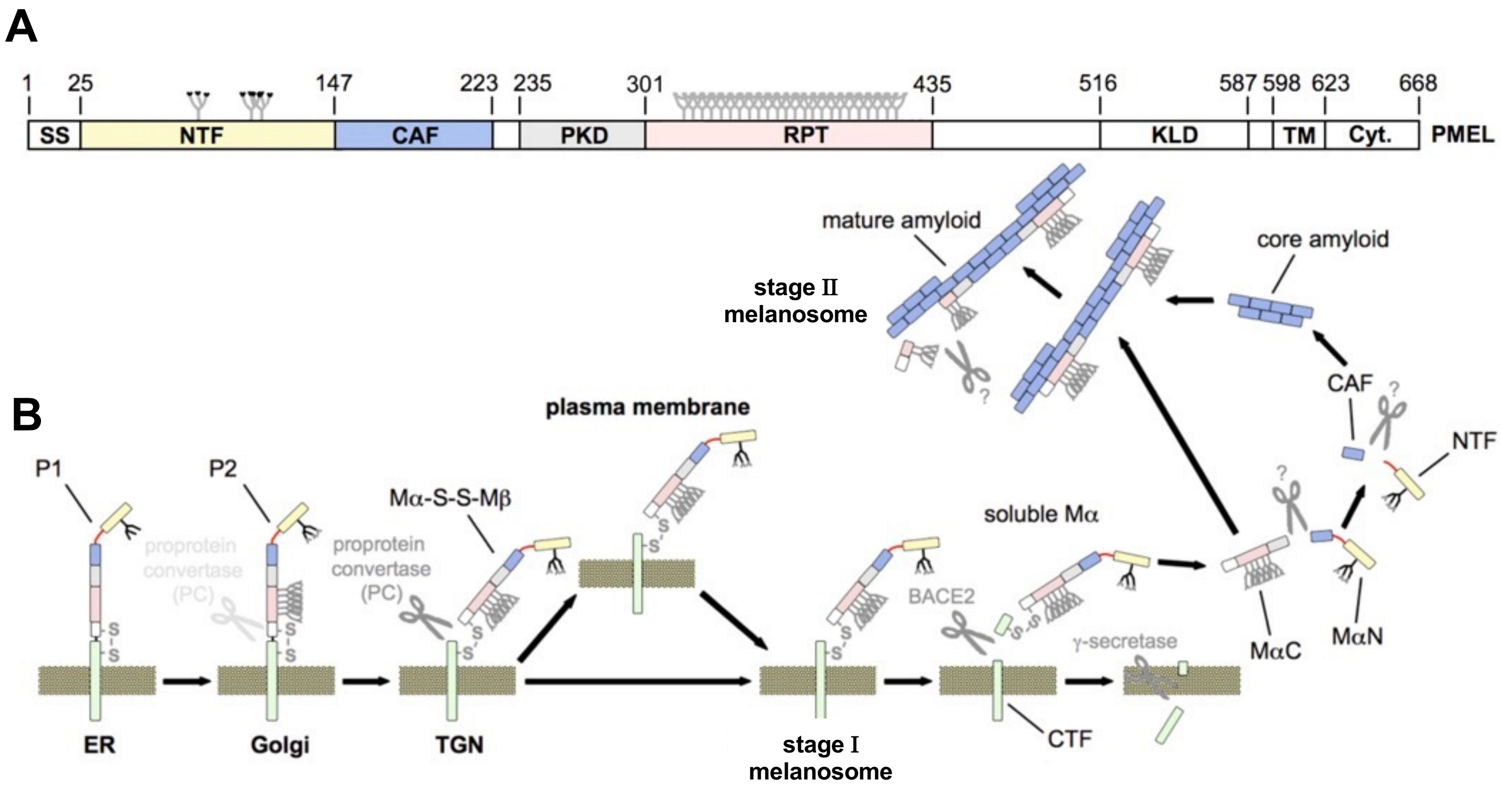

Supplementary Figure S1

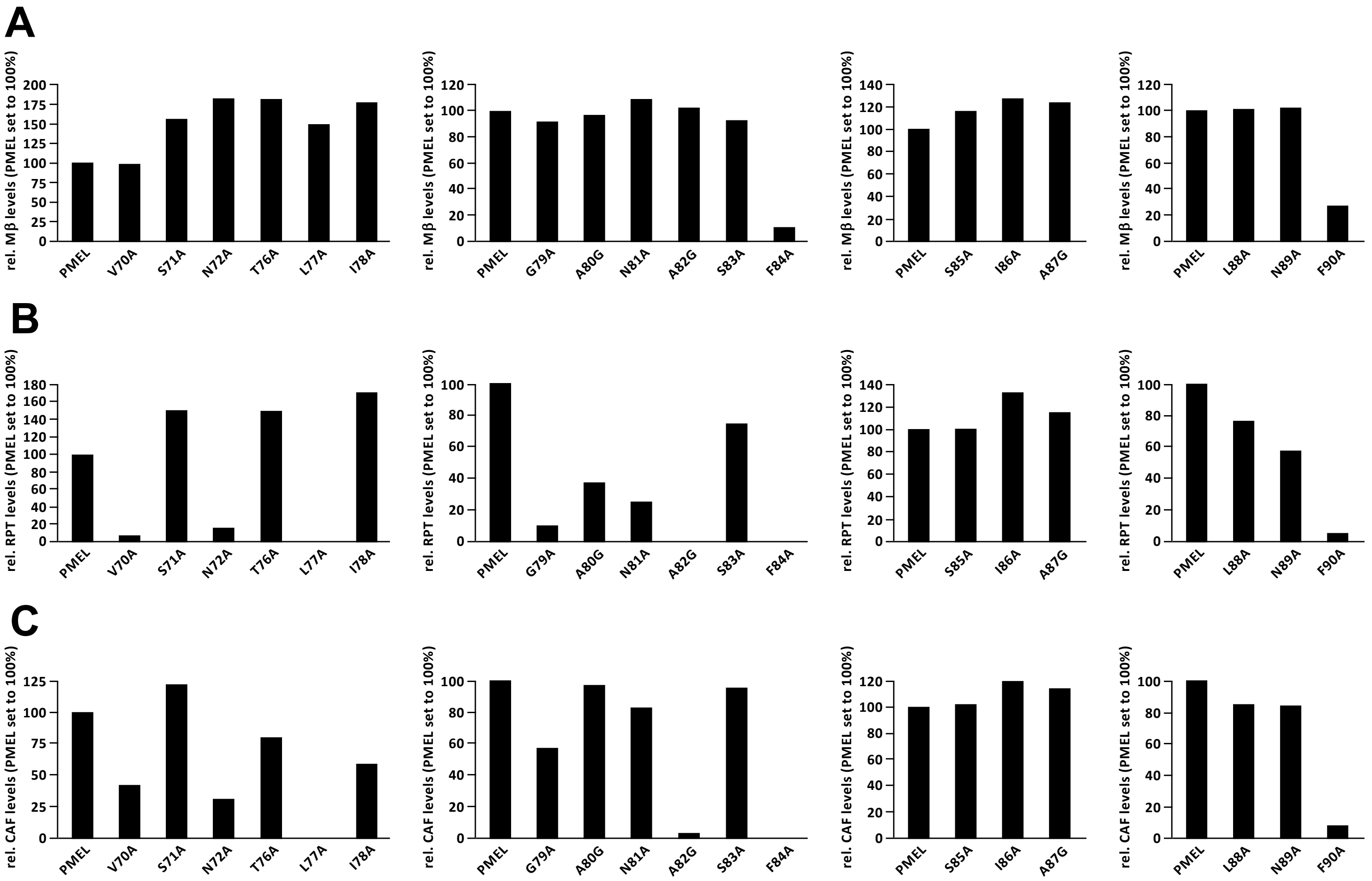

Supplementary Figure S2

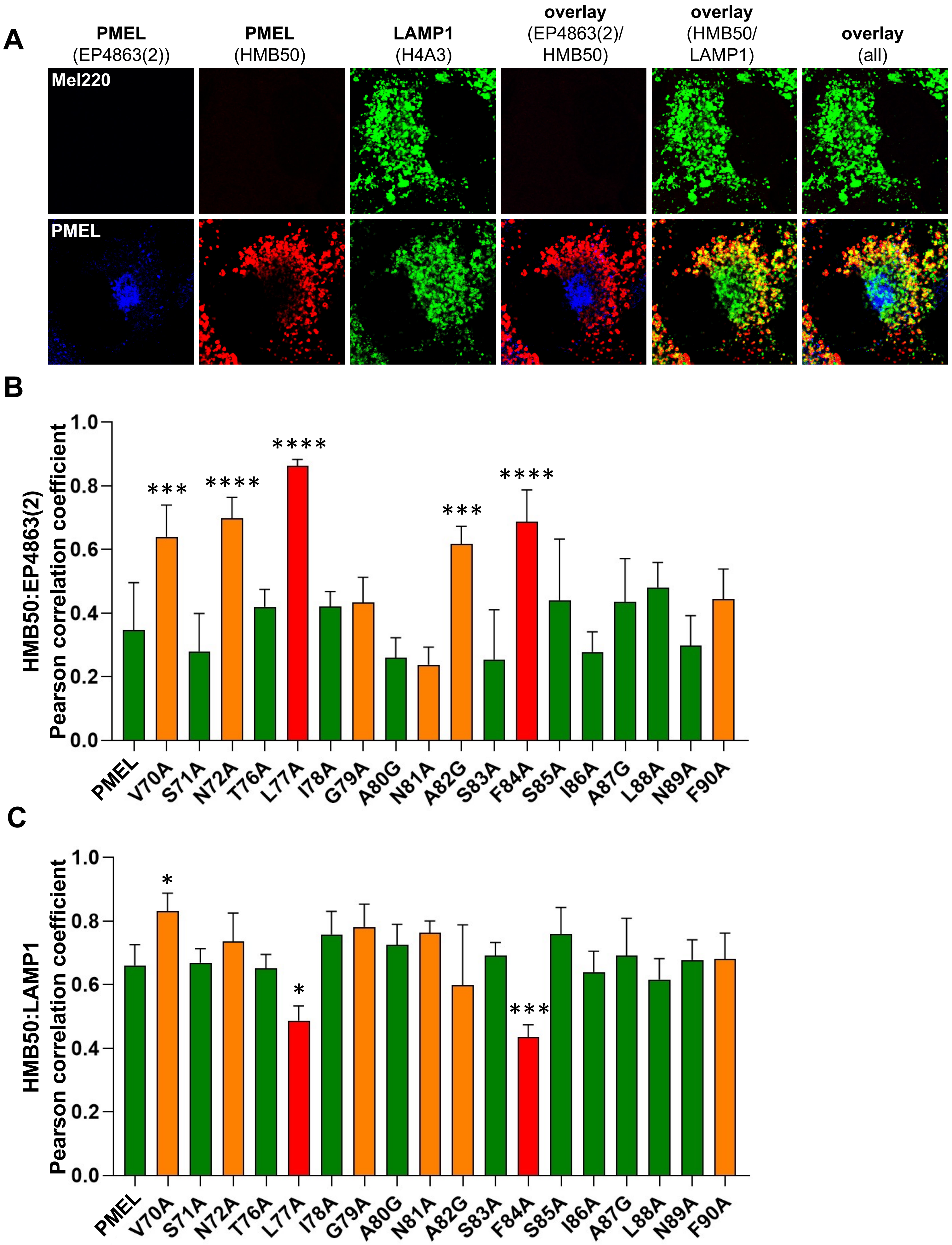

Supplementary Figure S3

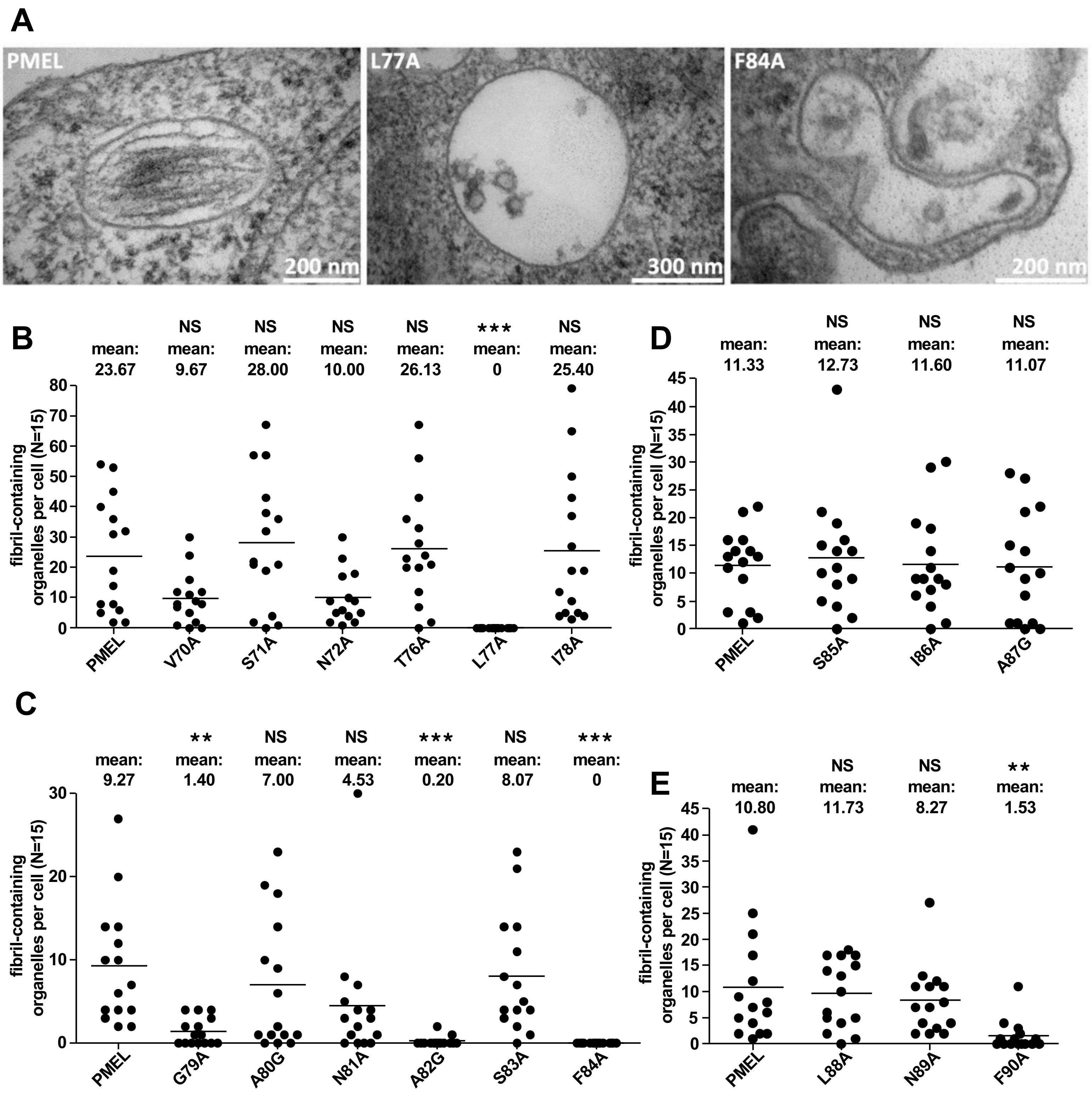

Supplementary Figure S4

A

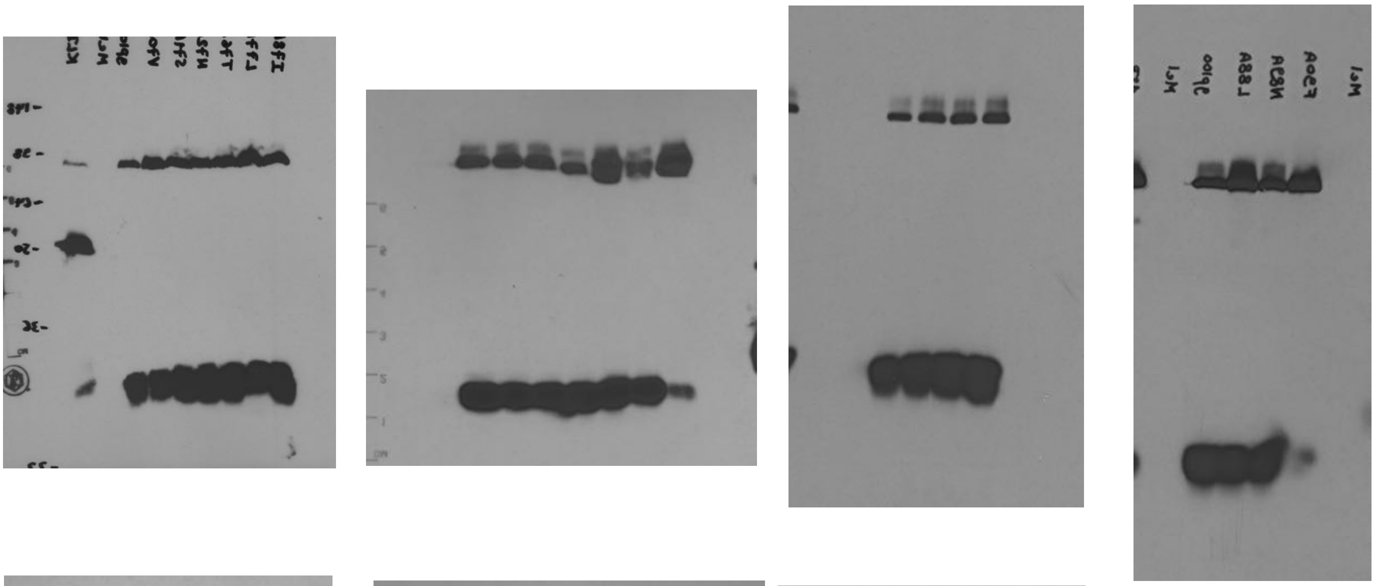

B

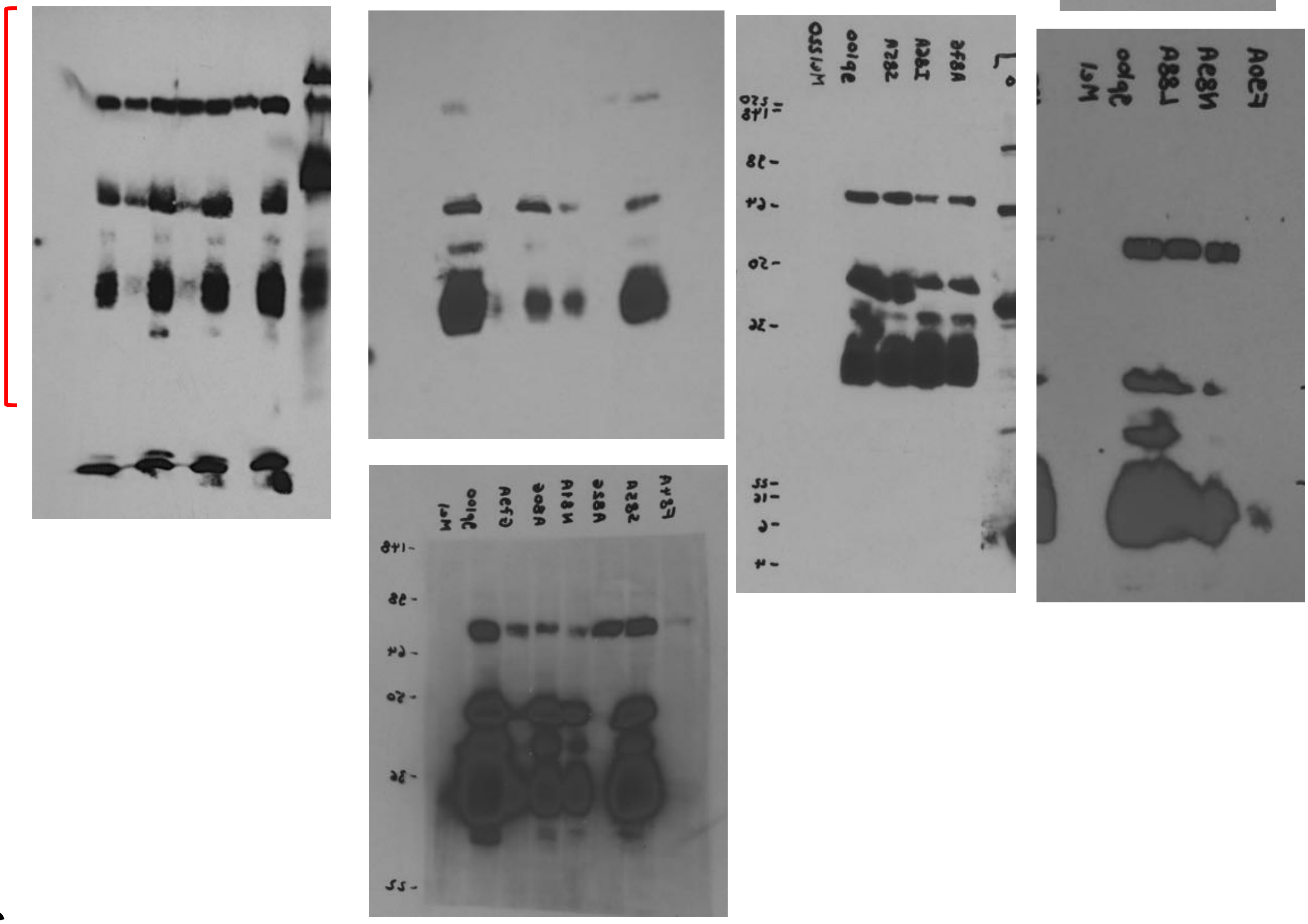

C

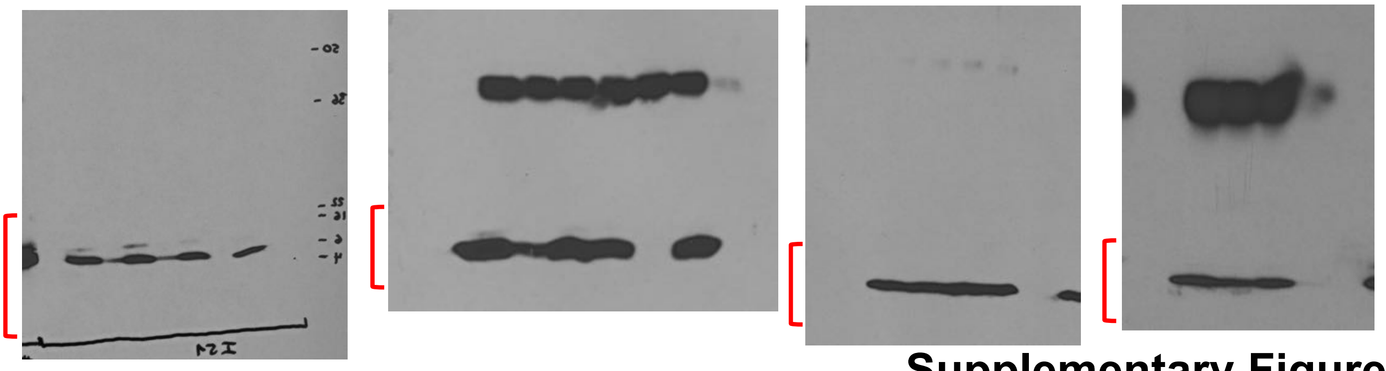

Supplementary Figure S5
